# *Scutellaria baicalensis* Attenuates Airway Remodeling via PI3K/Akt/NF-*κ*B Pathway in Cigarette Smoke Mediated-COPD Rats Model

**DOI:** 10.1155/2018/1281420

**Published:** 2018-05-13

**Authors:** Fei Xu, Jinpei Lin, Wenqiang Cui, Qing Kong, Qiuping Li, Lulu Li, Ying Wei, Jingcheng Dong

**Affiliations:** ^1^Department of Integrative Medicine, Huashan Hospital, Fudan University, Shanghai, China; ^2^Institutes of Integrative Medicine, Fudan University, Shanghai, China; ^3^Department of Integrative Medicine and Neurobiology, State Key Laboratory of Medical Neurobiology, Institute of Acupuncture Research, School of Basic Medical Science, Fudan University, Shanghai, China

## Abstract

*Background. Scutellaria baicalensis* (SB) is commonly used in traditional Chinese medicine for chronic inflammatory diseases. This study aims to investigate the effects of the early intervention with SB on airway remodeling in a well-established rat model of COPD induced by cigarette smoking.* Methods.* COPD model in Sprague Dawley (SD) rats were established by exposing them to smoke for 6 days/week, for 12 weeks, 24 weeks, or 36 weeks. Meanwhile, rats were randomly divided into normal control group, model group, Budesonide (BUD) group, and the SB (low, middle, and high) dose groups with 8 rats in each group and 3 stages (12 weeks, 24 weeks, and 36 weeks). After treatment, the pulmonary function was evaluated by BUXCO system and the morphology changes of the lungs were observed with HE and Masson staining. The serum IL-6, IL-8, and IL-10 and TNF-*α*, TGF-beta (TGF-*β*1), MMP-2, MMP-9, and TIMP-1 levels in BALF were detected by ELISA-kit assay. The protein expression levels of AKT and NF-*κ*B (p65) were determined by western blot (WB).* Results.* The oral of SB significantly improved pulmonary function (PF) and ameliorated the pathological damage and attenuated inflammatory cytokines infiltration into the lungs. Meanwhile, the levels of TGF-*β*, MMP-2, MMP-9, and TIMP-1 were partially significantly decreased. The levels of PI3K/AKT/NF-*κ*B pathway were also markedly suppressed by SB.* Conclusions.* SB could significantly improve the condition of airway remodeling by inhibiting airway inflammation and partially quenching TGF-*β* and MMPs via PI3K/AKT/NF-*κ*B pathway.

## 1. Introduction

Chronic obstructive pulmonary disease (COPD), characterized by presence of progressive and not fully reversible airflow obstruction, is a major cause of morbidity and mortality across the globe [1]. Even worse, World Health Organization (WHO) predicts that it will rise to the third leading cause by 2030 [2]. It is increasingly clear that remodeling of the peripheral airway wall in COPD is the core hallmark contributing to airflow limitation and involves airway and blood vessel factors [3, 4]. Risk factors have been actively investigated, and chronic exposure to tobacco smoke is the main trigger of inflammation in COPD [5]. Of note, evidence indicates that chronic inflammation is a causative factor leading to airway wall thickening, alveolar detachments, and reduction of airway-parenchyma interdependence leading to luminal narrowing [5, 6]. What is more, being exposed to proinflammatory agents frequently or for a long time may contribute to damage and metaplasia of the respiratory epithelium and exert angiogenic and antiangiogenic effects [7, 8]. Then activated epithelial cells transform into mesenchymal ones and meanwhile large quantities of cytokines/chemokines and metalloproteinases are expressed [9] and in turn aggravate lung injury, which form a vicious circle. A repair process in response to airway injuries resulting from sustained inflammation or mechanical stretch can stimulate the development of airway remodeling [10]. When COPD develops, an active and complex remodeling process is present in the peripheral lung [11] and the circle speeds up the formation of airway remodeling.

Throughout, airway remodeling is a dynamic process that is active and potentially progressive, but that can be prevented by appropriate therapy [12]. So far, managements involve long-acting bronchodilators (LB), comprised of long-acting beta2-agonists (LABA) and tiotropium, inhaled corticosteroid (ICS), phosphodiesterase, leukotriene antagonists, and so on [1, 13]. However, they can only reduce the number and severity of exacerbation and established symptoms and have a limited effect on slowing down the progression of lung damage, inflammation, and airway remodeling [14]. Furthermore, the use of these drugs is often accompanied by undesirable side effects.

In China, many years of clinic studies have demonstrated that* Scutellaria baicalensis* (SB) is frequently used in the treatment of influenza, cancer, oxidative activities, and chronic inflammatory diseases in the respiratory system in traditional Chinese medicine (TCM) [15]. Nevertheless, few articles have studied the effectiveness of SB treatment on airway remodeling of SB in COPD. Therefore, we aimed to study the effect of SB on airway remodeling of CS mediated-COPD rats model.

## 2. Materials Studies

### 2.1. Experimental Animals and Grouping

Male Sprague Dawley (SD) rats (8 weeks old, weighed 180–200 g) were purchased from s&p-Shall Kay Laboratory Animal Co., Ltd. (Shanghai, China), and then were housed at a constant temperature (23°C) under a 12 h light-dark cycle (lights on from 7:00 a.m. to 7:00 p.m.) and had free access to food and water. After one week of conditioning, a total of 140 male SD rats were randomly divided into 6 groups (*n* = 24 per group, 4 per cage): normal control group (NC group), COPD model group, SB low-dose group (1.5 mg/(kg·d)), SB middle-dose group (3 mg/(kg·d)), SB high-dose group (6 mg/(kg·d)), and Budesonide (BUD) group (0.2 mg/(kg·d)). The protocol has the approval of the Animal Experimental Ethical Committee of Fudan University and all animal studies were approved by the Institutional Review Board of Shanghai Medical College of Fudan University (permit number: SYXK (hu) 2010–0099) and in accordance with the guidelines for animal use of National Institutes of Health.

### 2.2. Tobacco Exposure

The experimental model of COPD was established by being exposed whole body to tobacco smoke 20 3R4F research cigarettes (Tobacco and Health Research Institute, University of Kentucky, KY) for 1 hour/day, 6 days/week, for either 12, 24, or 36 weeks. Three cigarettes for each challenge were smoked using a smoking machine and the cigarette smoking (CS) was consistently delivered into the chamber at a rate of approximately 15 min per cigarette; then open the lid and meanwhile let the rats rest for 5 minutes. Totally, rats were kept in the smoked chamber for 1 hour (h) in the morning and 1 h in the afternoon, 6 days a week, for 12, 24, and 36 weeks [16, 17]. Animals in control group underwent the same procedure but filtered air exposure. The amount of cigarette was increased with gradient to reach target dose.

### 2.3. Drugs and Delivery

SB was purchased from Chengdu Must Bio-Technology Co., Ltd. (Chengdu, China). Rats were dosed with SB (1.5 mg/(kg·d), 3 mg/(kg·d), and 6 mg/(kg·d)) by intragastric administration in 0.3 ml volume per day for six days 1 hour before exposure to cigarette smoke (CS). The control group was dosed with 0.9% of physiological saline at the same volume under the same procedure, while the COPD model group was treated with Budesonide 0.2 mg/(kg·d), as a positive control drug by using ultrasonic atomizing inhalation, and was administered just the same as NC group.

### 2.4. Pulmonary Function

Briefly, after the last exposure, rats were anesthetized with 2% pentobarbital sodium (1 ml/100 g ip). Then a tracheotomy was made and the tracheal tube was inserted with a suture around the trachea. Rats were put into the whole-body plethysmography chamber (Buxco, USA) for anesthetized animals and the inserted tracheal tube was connected to the ventilator. The use of this method permitted continuous monitoring of a number of ventilatory parameters, including the ratio of forced expiratory volume in the first 0.1 seconds (FEV0.1) and forced vital capacity (FVC) (FEV0.1/FVC%); maximal mid-expiratory flow (MMEF); and peak expiratory flow (PEF).

### 2.5. BALF Collection

After pulmonary function measurement, the lungs were then lavaged by inserting a cannula into the trachea with 460.3 ml aliquots of ice-cold sterile phosphate-buffered saline (PBS). All aliquots were combined for individual mice. The BALF was immediately centrifuged at 1500*g* for 10 min at 4°C, and the cell-free supernatant was stored at −80°C.

### 2.6. Blood Collection

Immediately after the BALF collection, blood collection was conducted from the orbital venous plexus; blood was stored at 4°C for 2 hours and then centrifuged at 5000 ×g at 4°C for 15 minutes. The serum was collected, repackaged, and stored at −80°C for ELISA assays.

### 2.7. Pulmonary Histopathology

The right lobe of the lung was removed, fixed in 4% paraformaldehyde for 24 hours, and embedded in paraffin for histopathology analysis. Lung tissues were cut into 2 um thick sections, stained with hematoxylin-eosin (HE) and Masson, and examined for airway inflammation changes by light microscopy.

To quantitate bronchial wall thickness, a modification of the approach once used by Han et al. [18] was applied. Firstly, diameter of 200 um in 3 bronchioles per lung section was randomly outlined and then area sizes were measured using MIQAS. The bronchial wall thickness was calculated as follows: smooth muscle area (WAsm) = the lateral area of the smooth muscle layer (Asmo) − the medial area of the smooth muscle layer (Asmi). Tube wall perimeter (Pi) was standardized. The results in the smooth muscle layer area per unit length (WAsm/Pi, um^2^/um) represent the smooth muscle layer thickness. Similarly, to quantitate the thickness of pulmonary arteriolar smooth muscle (PASM), the above methods were employed.

In addition, to evaluate to the mean linear intercepts (MLI), we instead assessed the change of air space size by a modified procedure once used by Sato et al. [19]. Firstly, we randomly selected fields in each section at ×100 magnification and then calculated the numbers of alveolar septum in the area. The destructive index (DI) was calculated using the method described by Saetta et al. [20] of determining the destruction of the alveolar wall. Three randomly selected fields in each section at ×100 magnification were used to measure DI.

### 2.8. Cytokine Analysis

IL-6, IL-8, and IL-10 in serum and TNF-*α*, TGF-*β*1, MMP-2, MMP-9, and tissue inhibitor of metalloproteinase-1 (TIMP-1) levels in BALF were measured by ELISA-kit according to the manufacturer's instructions.

### 2.9. Western Blot (WB)

Western blotting was performed as previously reported [21], and quantitative analyses were performed using Image J software.

### 2.10. Statistical Analysis

The results were expressed as mean ± standard deviation. Statistical analyses were performed using analysis of variance or Student's *t*-tests. Data were undertaken using the SPSS18.0 package and a *P* value less than 0.05 was considered statistically significant.

## 3. Results

### 3.1. Effects of SB on Pulmonary Function in CS-Exposed Rats

**Figure 1:**
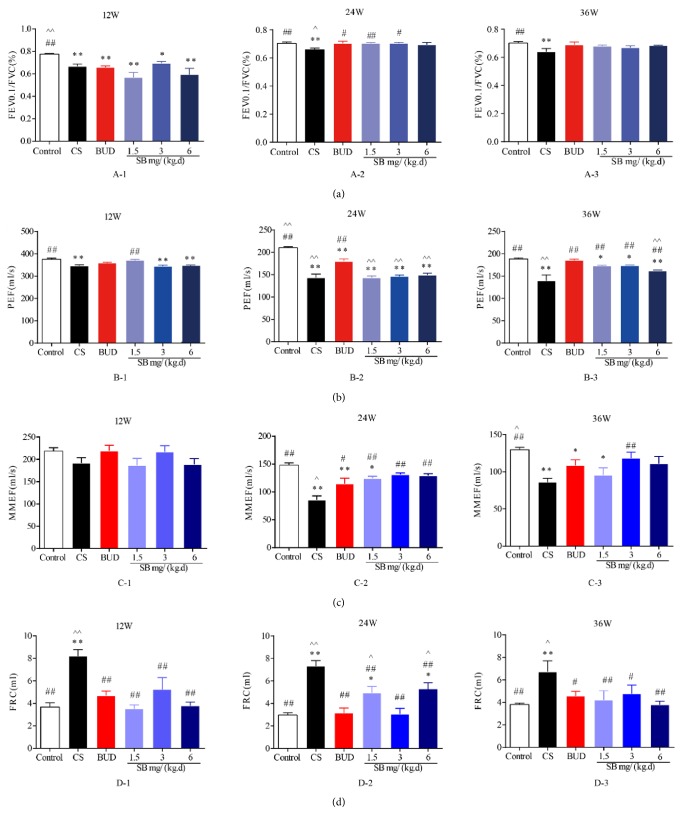
The effect of SB on pulmonary function in CS-exposed rats of different stages. Rats were exposed to CS for 12 weeks, 24 weeks, and 36 weeks with SB (1.5, 3, and 6 mg/(kg·d)) or BUD (0.2 mg/(kg·d)). Pulmonary function: FEV0.1/FVC (%) (a), PEF (b), MMEF (c), and FRC (d) were measured which indicated the model of COPD was successfully established. Data are mean ± SEM (*n* = 8). ^*∗*^*P* < 0.05, ^*∗∗*^*P* < 0.01, compared with the normal control group. ^#^*P* < 0.05, ^##^*P* < 0.01, compared with the COPD model group. ^∧^*P* < 0.05, ^∧∧^*P* < 0.05, compared with BUD group. CS = cigarette smoking; SB =* Scutellaria baicalensis*; BUD = Budesonide; FEV0.1 = forced expiratory volume in the first 0.1 seconds; FVC = forced vital capacity; PEF = forced expiratory flow; MMEF = maximum mid expiratory flow rate; and FRC = functional residual capacity.

Lung function test showed that FEV0.1/FVC (%), PEF, and MMEF were significantly lower in the groups with COPD than the normal control groups (*P* < 0.01, Figures 1(a), 1(b), and 1(c)), while FRC of the COPD groups was significantly higher than that of the normal control groups (*P* < 0.01, Figure 1(d)). SB showed improvement of lung function between the normal groups and the model groups, and the differences were statistically significant (*P* < 0.05 or *P* < 0.01, Figures 1(a), 1(b), and 1(c)). Whereas rats treated with SB showed significant (*P* < 0.05, Figure 1) growth on FEV0.1/FVC, PEF, and MMEF (Figures 1(a), 1(b) and 1(c)), they showed decrease on FRC (Figure 1(d)) compared with CS-exposed rats in time-dependent manner. High dose of SB had better effect than both low-dose and middle-dose. Besides, FEV0.1/FVC and FRC in the model groups made no difference compared to BUD groups (Figures 1(a) and 1(d)). Nevertheless, when opposed to BUD, SB showed more promising effect as time went by.

### 3.2. Effects of SB on Lung Histological Examination

**Figure 2:**
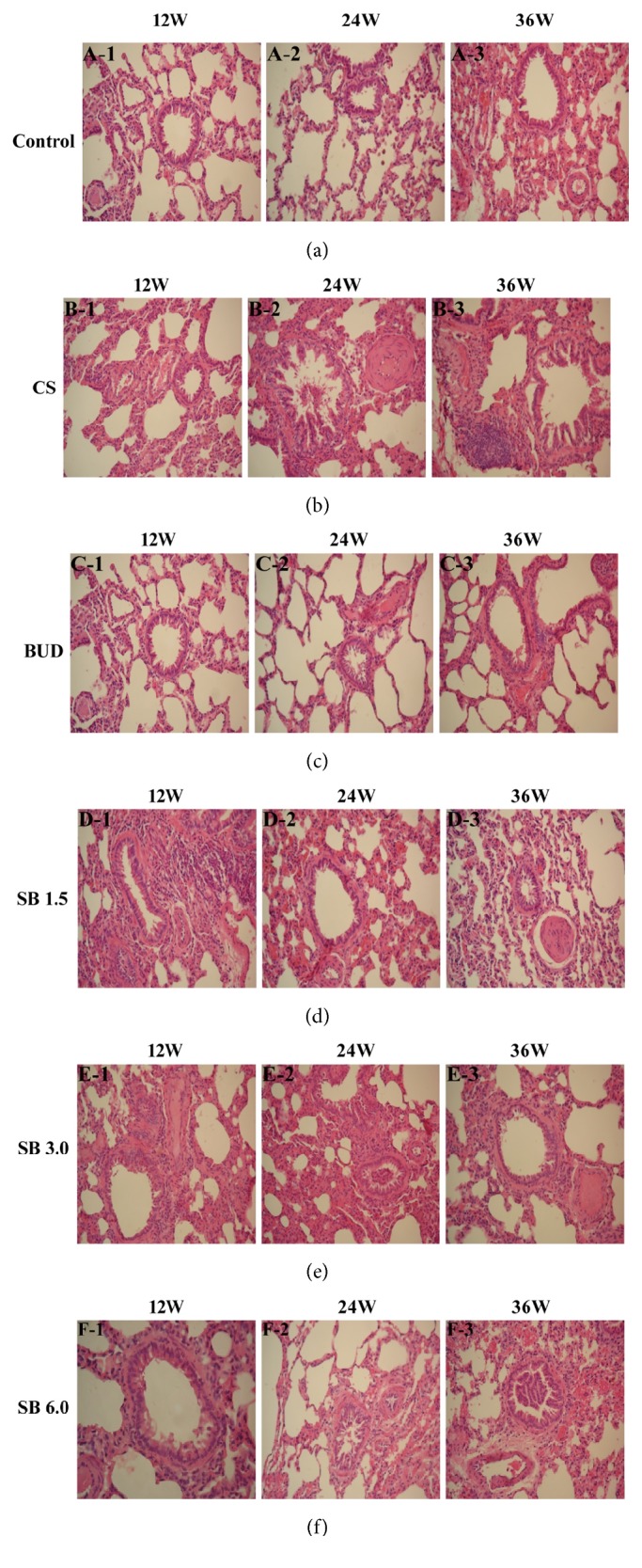
The effect of SB on histological examination by HE staining. Rats were exposed to CS for 12, 24, and 36 weeks with SB (1.5, 3, and 6 mg/(kg·d)) or BUD (0.2 mg/(kg·d)). Lung tissue was stained using H&E being examined. (a) NC group; (b) COPD model group; (c) BUD group; (d) SB low-dose group; (e) SB middle-dose group; and (f) SB high-dose group. Original magnification, ×200. CS = cigarette smoking; HE = hematoxylin and eosin staining; SB =* Scutellaria baicalensis*; BUD = Budesonide; and NC = normal control.

**Figure 3:**
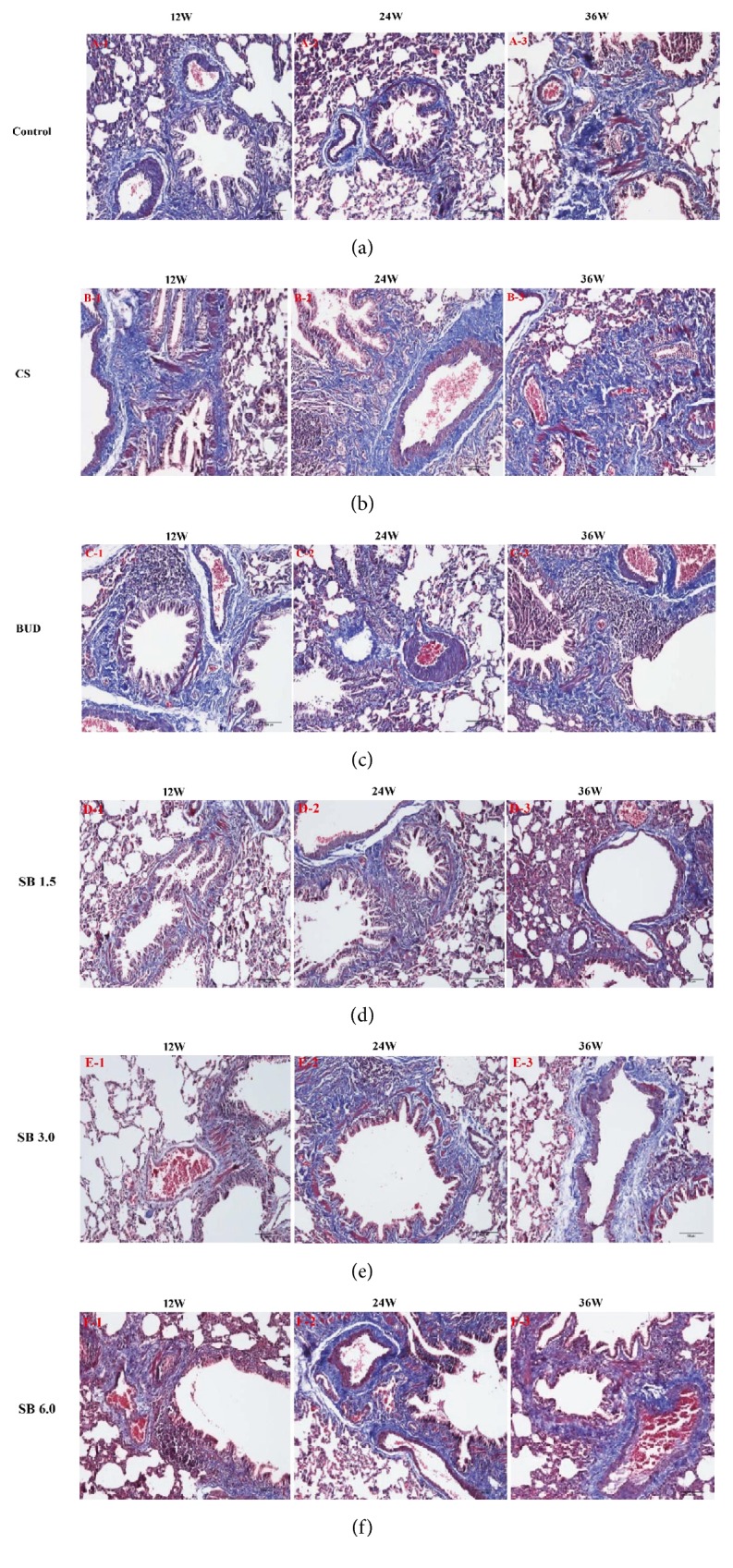
The effect of SB on histological analysis revealed typical pathological features by Masson staining. Pictures presented the histology of lung by Masson at three stages, 12 weeks, 24 weeks, and 36 weeks, respectively. (a) NC group; (b) COPD model group; (c) BUD group; (d) SB low-dose group; (e) SB middle-dose group; and (f) SB high-dose group. Original magnification, ×200. CS = cigarette smoking; SB =* Scutellaria baicalensis*; BUD = Budesonide; and NC = normal control.

Compared to normal groups, the histological analysis of lung tissue of the model groups presented the following features: after 12 weeks obvious inflammatory cells infiltration and notable edema; abundance of goblet cells, extensive areas of mucous secretions, and inflammatory cells within the lumen of the small bronchial airway; extensive areas of epithelial metaplasia and loss along the central bronchial airway; alveolar expansion, rupture, and fusion; significantly thicker pulmonary arterial wall and so on (Figures 2(a), 2(b), 3(a) and 3(b)). SB treatment can help improve the typical pathological features of COPD. As shown in HE and Masson staining, compared to the model groups, airway inflammation and edema, goblet cell hyperplasia, and alveolar expansion and fusion were reduced more greatly at 12 weeks with every dose (Figures 2 B-1, D-1, E-1, and F-1 and Figures 3 B-1, D-1, E-1, and F-1). Additionally, at 24 and 36 weeks, compared with the normal groups, chronic bronchitis and obstructive emphysema were observed in low-dose groups, but inflammatory cell infiltration was significantly decreased (Figures 2 A-2, A-3, D-2, and D-3). Administration of middle and high dosage of SB resulted in a significant decrease of the lung inflammation score after 12 weeks compared with other groups, even much better than BUD groups (Figures 2 C-1, E-1, and F-1); however, small pulmonary arteries and small bronchial smooth muscle layer showed marked thickness, including the presence of bronchial lumen stenosis (Figures 2 C-1, E-1, and F-1).

**Figure 4:**
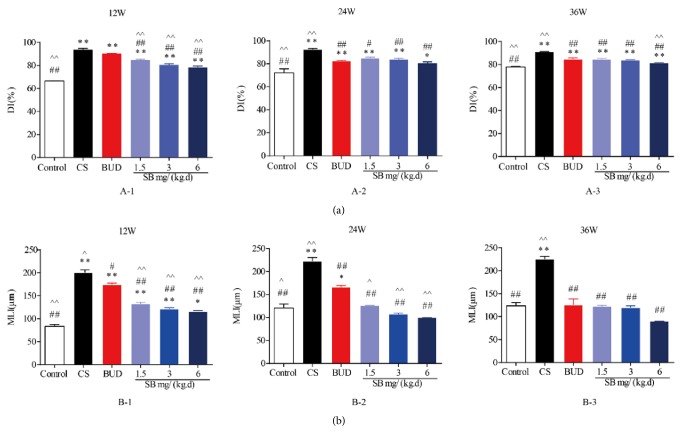
The effect of SB on MLI and DI. Values of MLI and DI were calculated as described in Materials and Methods. Data are mean ± SEM (*n* = 8). ^*∗*^*P* < 0.05, ^*∗∗*^*P* < 0.01, compared with the normal control group. ^#^*P* < 0.05, ^##^*P* < 0.01, compared with the COPD model group. ^∧^*P* < 0.05, ^∧∧^*P* < 0.05, compared with BUD group. SB =* Scutellaria baicalensis*; BUD = Budesonide; MLI = mean linear intercepts; and DI = destructive index.

In addition, our data showed that every dosage of SB can effectively reduce both MLI and DI in dose-dependent manner, which displayed significant differences between CS and normal groups (*P* < 0.05 or *P* < 0.01, Figures 4(a) and 4(b)). Furthermore, at 12 weeks there was a significant difference between SB- or BUD-treated rats and the normal groups (*P* < 0.01, Figures 4 A-1 and B-1), which indicated that SB can improve the degree of lung parenchyma injury and are superior to BUD. What is more, in the course of the experiment, SB slowed down the progress of emphysema.

### 3.3. Effects of SB on Airway Remodeling Assessment

**Figure 5:**
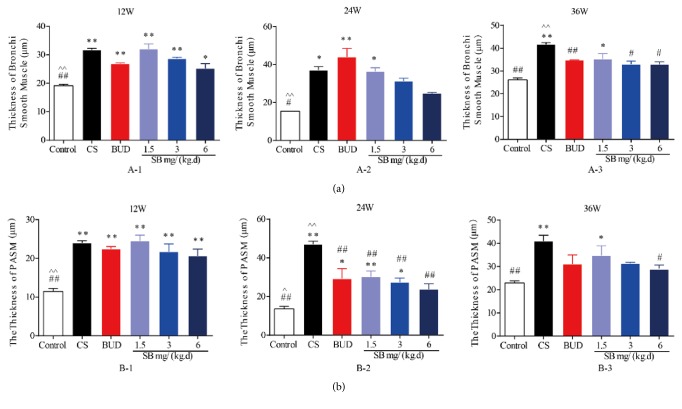
The effect of SB on airway remodeling assessment. Rats were exposed to CS for 12, 24, and 36 weeks with SB (1.5, 3, and 6 mg/(kg·d)) or BUD (0.2 mg/(kg·d)). Lung tissue was stained using HE and Masson before being calculated. The thickness of bronchial smooth muscle and PASM in 200 um was quantitated. Data are mean ± SEM (*n* = 3). ^*∗*^*P* < 0.05, ^*∗∗*^*P* < 0.01, compared with the normal control group. ^#^*P* < 0.05, ^##^*P* < 0.01, compared with the COPD model group. ^∧^*P* < 0.05, ^∧∧^*P* < 0.05, compared with BUD group. CS = cigarette smoke; SB =* Scutellaria baicalensis*; BUD = Budesonide; HE = hematoxylin and eosin staining; PASM = pulmonary arteriolar smooth muscle.

As mentioned above, HE and Masson staining pathological section of lung tissue in model groups showed a progressive process of airway inflammation and remodeling. The thickness of bronchial smooth muscle and PASM in 200 um and the model groups were significantly higher than the normal groups (*P* < 0.05 or *P* < 0.01, Figures 5(a) and 5(b)). At 36 weeks, SB in every dose group could improve the thickening of bronchial smooth muscle and PASM in a dose-dependent manner (Figure 5) and but only the high-dose group had the equal effect to BUD group and had a significant difference (*P* < 0.05, Figure 5) compared with the model group.

### 3.4. Effects of SB on Level of CS-Induced IL-6, IL-8, and IL-10 in Serum

**Figure 6:**
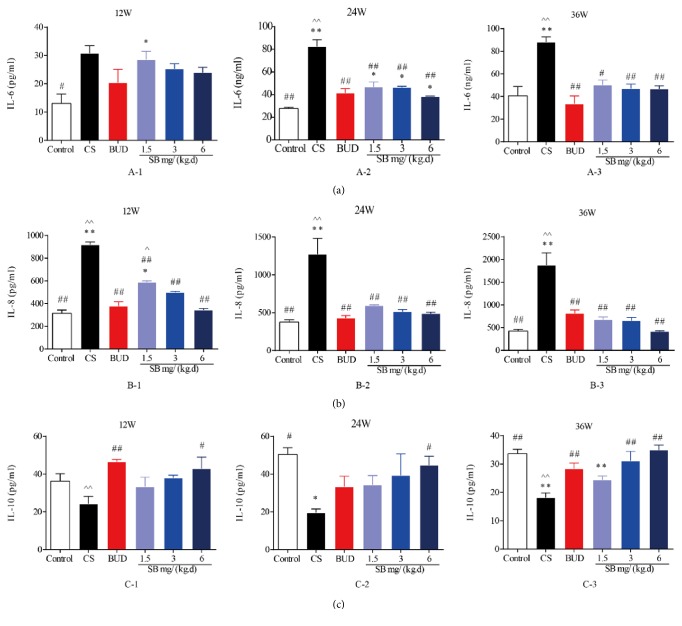
The effect of SB on inflammatory factor. IL-8 and IL-10 were measured by ELISA assays. Rats were exposed to CS for 12, 24, and 36 weeks with SB (1.5, 3, and 6 mg/(kg·d)) or BUD (0.2 mg/(kg·d)). Data are mean ± SEM (*n* = 8). ^*∗*^*P* < 0.05, ^*∗∗*^*P* < 0.01, compared with the normal control group. ^#^*P* < 0.05, ^##^*P* < 0.01, compared with the COPD model group. ^∧^*P* < 0.05, ^∧∧^*P* < 0.05, compared with BUD group. CS = cigarette smoke; SB =* Scutellaria baicalensis*; BUD = Budesonide; and IL = interleukin.

To further characterize the effects of SB on airway modeling by modulating inflammatory response to CS exposure, we analyzed some inflammatory cytokines, proinflammatory cytokine IL-6, IL-8, and anti-inflammatory cytokine IL-10. As shown in Figure 6, compared with normal group, the level of IL-6 and IL-8 in the model group significantly increased (*P* < 0.01), but IL-10 decreased (*P* < 0.01) in time-dependent manner. Compared with the model group, the serum level of IL-6 and IL-8 in BUD group decreased significantly (*P* < 0.01, Figures 6(a) and 6(b)), while the serum level of IL-10 increased significantly (*P* < 0.05 or *P* < 0.01, Figure 6(c)). The same as BUD groups, SB were able to decrease IL-6 and IL-8 serum level as dosage increased; in contrast, the level of IL-10 increased as dosage increased (*P* < 0.05 or *P* < 0.01, Figure 6). Changes to the serum level of IL-6, IL-8, and IL-10 at the middle and high dosage were all statistically significant (*P* < 0.05 or *P* < 0.01, Figure 6). However, there was almost no significance between SB and BUD groups (*P* > 0.05, Figure 6).

### 3.5. Effects of SB on Level of CS-Induced TNF-*α*, TGF-*β*1, MMP-2, MMP-9, and TIMP-1 Level in BALF

**Figure 7:**
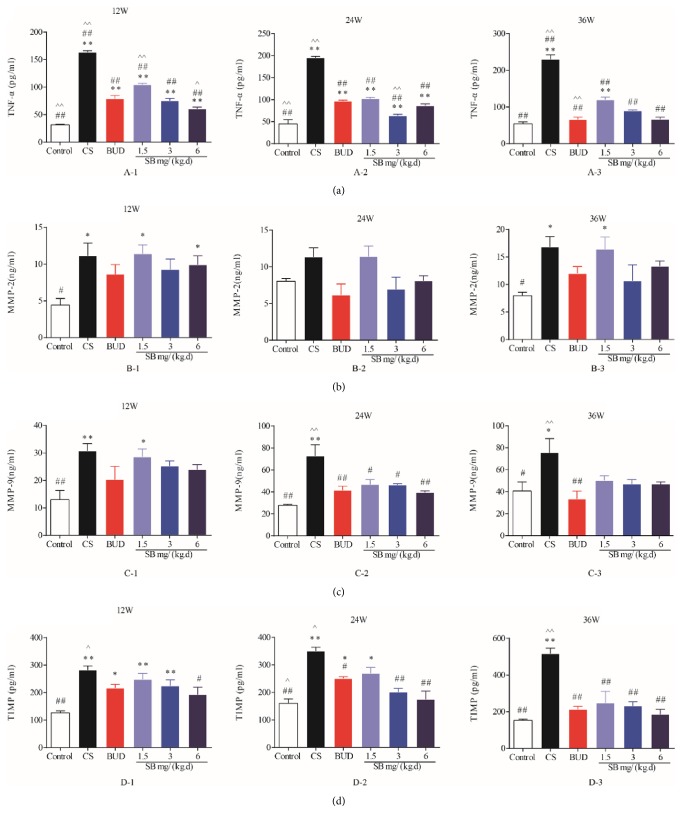
The effect of SB on cytokines, TGF-*β*1, MMP-2, MMP-9, and TIMP-1. Rats were exposed to CS for 12, 24, and 36 weeks with SB (1.5, 3, and 6 mg/(kg·d)) or BUD (0.2 mg/(kg·d)). Levels of TGF-*β*1, MMP-2, MMP-9, and TIMP-1 were determined by ELISA. Data are mean ± SEM (*n* = 8). ^*∗*^*P* < 0.05, ^*∗∗*^*P* < 0.01, compared with the normal control group. ^#^*P* < 0.05, ^##^*P* < 0.01, compared with the COPD model group. ^∧^*P* < 0.05, ^∧∧^*P* < 0.05, compared with BUD group. CS = cigarette smoke; SB =* Scutellaria baicalensis*; BUD = Budesonide; MMP = matrix metalloproteinase; and TIMP = tissue inhibitor of metalloproteinase.

Compared with the normal control group, the BALF levels of TNF-*α*, TGF-*β*1, MMP-2, MMP-9, and TIMP-1 in the model group significantly increased (*P* < 0.05 or *P* < 0.01, Figure 7) as time went on. Compared with the model group, the levels of TNF-*α*, TGF-*β*1, MMP-2, MMP-9, and TIMP-1 in BALF were significantly decreased in the BUD group (*P* < 0.05 or *P* < 0.01, Figure 7), but the levels of TGF-*β*1, MMP-2, MMP-9, and TIMP-1 in SB groups gradually reduced to some degree and as dosage increased (*P* < 0.05 or *P* < 0.01, Figure 7). What is more, changes to these cytokines except MMP-2 and TNF-*α* (at 36 weeks) at highest dosage were all statistically significant (*P* < 0.01, Figure 7 A-1) compared with the model groups, but they all were partially better than those of BUD group (*P* < 0.05 or *P* < 0.01, Figure 7), whereas the highest efficacy of SB to MMP2 is at middle dosage (24 mg/(kg·d), Figure 7(b)).

### 3.6. Effects of SB on Levels of PI3K/AKT/NF-*κ*B in CS-Mediated-COPD Rats Model

**Figure 8:**
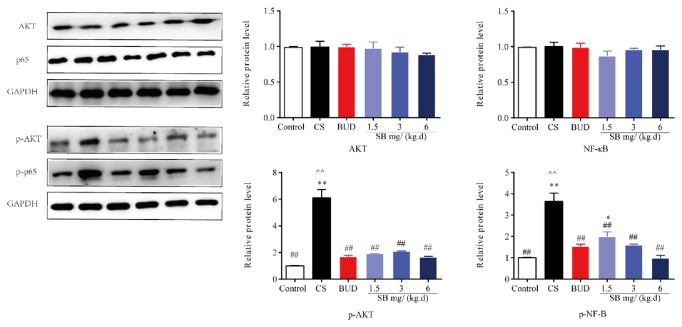
The effect of SB on PI3K/AKT/NF-*κ*B pathway. Rats were exposed to CS for 24 weeks with SB (1.5, 3, and 6 mg/(kg·d)) or BUD (0.2 mg/(kg·d)). Levels of AKT, p-AKT, NF-*κ*B (p65), and p-NF-*κ*B (p-p65) were determined by western blot (WB). Data are mean ± SEM (*n* = 8). ^*∗*^*P* < 0.05, ^*∗∗*^*P* < 0.01, compared with the normal control group. ^#^*P* < 0.05, ^##^*P* < 0.01, compared with the COPD model group. ^∧^*P* < 0.05, ^∧∧^*P* < 0.05, compared with BUD group. CS = cigarette smoke; SB =* Scutellaria baicalensis*; and BUD = Budesonide.

In our experiment, we confirmed the presence of airway remodeling in the lung of rats with COPD. The airway remodeling reaches its peak at 24 weeks' inhalation of cigarette smoke. Then the signaling pathway was conducted at 24 weeks. To further elucidate whether the PI3K/AKT/NF-*κ*B signaling pathways participated in the regulation of airway remodeling, western blot analysis was used to determine their expression levels. The results showed that there was a significant increase in AKT and NF-*κ*B phosphorylation (p-AKT and p-NF-*κ*B) in the lungs of mice exposed to CS for 24 weeks as compared with controls (*P* < 0.01, Figure 8). Interestingly, BUD and SB succeeded to cause significant suppression of p-AKT and p-NF-*κ*B compared with CS-exposed mice (*P* < 0.01, Figure 8). However, no significant differences were noted in protein levels of p-AKT and p-NF-*κ*B between the BUD and SB groups (*P* > 0.05, Figure 8).

## 4. Discussion

In this study, we investigated the anti-airway remodeling effects of SB in cigarette smoke induced models in rats, which was likely achieved by modulating PI3K/AKT/NF-*κ*B pathway.

COPD is a chronic inflammatory disease, along with two fundamental features: airway remodeling and emphysema [22]. Airway remodeling was highly related to airflow obstruction in COPD, characterized by small airway thickening, metaplasia of the epithelium, subepithelial fibrosis, increase in goblet cell size, increased accumulation of smooth muscle bundles and extracellular matrix (ECM), airway wall edema, and smooth muscle hypertrophy/hyperplasia [23–26]. CS, which drives an inflammatory response, is strongly relevant to the development of COPD [27–29]. In this study, results from decline in lung function parameters, airway enlargements and remodeling, histological analysis of lung tissue infiltrated inflammatory cells and edema, increasing thickness of smooth muscle in bronchus and PASM, and pathology of emphysema confirmed that the rat model of COPD was successfully established.

There is an agreement that chronic inflammation is important for damage and metaplasia of the respiratory epithelium [9]. Exposure to inflammation for prolonged periods of time results in connective tissue deposition in the airway walls [30]. Furthermore, previous reports have identified that epithelium produces epidermal growth factors (EGFs) and various proteases, mainly MMPs, which inhibit the degradation of extracellular matrix (ECM), whose changes in composition and quantity are associated with wall thickening [31]. CS contains copious amounts of chemical compounds, which have the capacity to induce chronic inflammation and damage to the airways. Interestingly, longstanding inflammation contributes to structural and cellular damage, which lays a foundation of fibrosis and obliteration of small airways, forming a vicious circle [32, 33]. Additionally, cytokines were responsible for airway inflammation in COPD. In line with previous research results [34, 35], we have shown that inflammatory cytokines, such as IL-6, IL-8, and TNF-*ɑ*, were markedly increased, while anti-inflammatory cytokine IL-10 was decreased (Figure 6) in response to CS. Furthermore, in accordance with previous studies [32], CS exerts an effect on endothelial cell damage and dysfunction, which initiates the sequence of events resulting in ECM deposition by altering the profile of matrix proteins released aggravating airway remodeling [36–39]. Consistent with these studies, our results also indicated that TGF-*β*, MMPs, and TIMP were highly elevated in COPD model (Figure 7), which play a pivotal role in the airway remodeling.

SB, one kind of traditional Chines herbal, has been increasingly used in the last decades and become well known for its significant role in preventing and treating inflammatory disease and cancer [40, 41]. In the present study, the decline in FEV0.1/FVC (%), PEF, and MMEF and the rise in FRC in response to CS were normalized by SB, supporting the ability of SB on alleviation of airway obstruction and pulmonary function. However, there was almost no significant difference between BUD and SB groups, which means that SB can protect lung function of rats from smoke exposure, to some extent. Moreover, SB significantly decreased inflammatory infiltration in both BALF and serum, MLI, DI, bronchial smooth muscle, and PASM in CS-exposed rats and inhibited the production of TGF-*β*, MMPs, and TIMP. Collectively, our data demonstrated that SB could ameliorate the typical pathological features of COPD, airway remodeling.

Previous studies have reported that the anti-inflammatory and fibrosis effects of baicalin, an extract of SB, were mediated through one or more NF-*κ*B signaling pathways in other diseases [35, 42]. More importantly, NF-*κ*B is a star molecule regulating inflammatory pathway. However, it was unclear whether PI3K/AKT/NF-*κ*B activation mediates CS induced airway remodeling in COPD rats. In this study, we investigated the effect of SB on the activation of this pathway induced by CS in COPD models. It is worthy mentioning that SB treatment significantly blocked the activation of the PI3K/AKT/NF-*κ*B pathways.

In the current study, we have demonstrated that SB, a kind of traditional Chinese medicine, can attenuate airway remodeling of CS mediated-COPD rats model by decreasing the airway inflammation, thickness of airway and small pulmonary arterioles wall, MLI and DI in CS-exposed rats, and suppressing the production of inflammation mediators. What is more, the results of ameliorating pulmonary function on increment in FEV0.1/FVC, PEF, and MMEF, but reduction in FRC by administration of SB, support the property of SB for reversing airway obstruction, meanwhile improving the condition of airway remodeling.

## 5. Conclusion

In conclusion, we have shown that SB has anti-airway remodeling effect on cigarette smoke induced inflammatory models in animal. Our data also provided further support for the critical role that SB plays in restoring the balance of proinflammation and anti-inflammation and recovering the ECM deposition/degradation imbalance by modulating PI3K/AKT/NF-*κ*B signal pathway.

## Supplementary Materials

Supplementary Materials Highlight about chronic obstructive pulmonary disease.
