# Supplementary material for: Scutellaria baicalensis Attenuates Airway Remodeling via PI3K/Akt/NF-κB Pathway in Cigarette Smoke Mediated-COPD Rats Model
Source: Evid Based Complement Alternat Med. 2018 May 13;2018:1281420. doi: 10.1155/2018/1281420 (PMC5971322; doi:10.1155/2018/1281420)
Supplement: Supplementary Materials — Highlight about chronic obstructive pulmonary disease. [file 1281420.f1.pdf]

As we all know, chronic obstructive pulmonary disease (COPD) is a major worldwide health problem, significantly decreasing life quality. Althought western medcine has effects on it, the side effects can not be ignored. Scutellaria baicalensis (SB) is frequently used in the treatment of influenza, cancer, oxidative activities and chronic inflammatory diseases in the respiratory system in traditional Chinese medicine. Our data provided further support for the critical role that SB plays in restoring the balance of pro-inflammation and anti-inflammation and recovering the ECM deposition/degradation imbalance by modulating PI3K/AKT/NF-κB signal pathway.
